# Homozygosity mapping identified a novel protein truncating mutation (p.Ser100Leufs*24) of the *BBS9* gene in a consanguineous Pakistani family with Bardet Biedl syndrome

**DOI:** 10.1186/s12881-016-0271-9

**Published:** 2016-02-04

**Authors:** Muzammil Ahmad Khan, Sumitra Mohan, Muhammad Zubair, Christian Windpassinger

**Affiliations:** Gomal Centre of Biochemistry and Biotechnology, Gomal University Dera Ismail Khan, Khyber-Pakhtoonkhwa, Khyber-Pakhtoonkhwa 29050 Pakistan; Institute of Human Genetics, Medical University of Graz, Graz, 8010 Austria; Interim Translational Research Institute, Genomic Core Facility, Academic Health System, Hamad Medical Corporation, Doha, 3050 Qatar

**Keywords:** BBS syndrome, Consanguinity, SNP microarray, Homozygosity mapping, *BBS9* gene, Protein truncation, PTHB1 domain

## Abstract

**Background:**

Bardet Biedl Syndrome (BBS) is a rare condition of multi-organ dysfunction with characteristic clinical features of retinal degeneration, truncal obesity, postaxial polydactyly, genital anomaly, intellectual disability and renal dysfunction. It is a hetero-genetic disorder and nineteen BBS genes have been discovered so far.

**Methods:**

Whole genome SNP genotyping was performed by using CytoScan® 750 K array (Affymetrix). Subsequently, the segregation of the disease locus in the whole family was carried out by genotyping STS markers within the homozygous interval. Finally, the mutation analysis was performed by Sanger DNA sequencing.

**Results:**

In the present molecular study a consanguineous Pakistani family, with autosomal recessive BBS, was analyzed. The clinical analysis of affected individuals presented with synpolydactyly, obesity, intellectual disability, renal abnormality and retinitis pigmentosa. The presented phenotype was consistent with the major features of BBS syndrome. Homozygosity mapping identified a common homozygous interval within the known BBS9 locus. Sequence analysis of *BBS9/PTHB1* gene revealed a single base deletion of c.299delC (p.Ser100Leufs*24) in exon 4. This frame-shift mutation presumably leads to a 122 amino acid truncated protein with complete loss of its C-terminal PTHB1 domain in combination with a partial loss of the N-terminal PTHB1 domain as well. *BBS9/PTHB1* gene mutations have been shown to be associated with BBS syndrome and to the best of our knowledge this study reports the first Pakistani family linked to the *BBS9* gene.

**Conclusion:**

Our molecular findings expand the mutational spectrum of *BBS9* gene and also explain the genetic heterogeneity of Pakistan families with BBS syndrome. The growing number of mutations in BBS genes in combination with a detailed phenotypical description of patients will be helpful for genotype-phenotype correlation, targeted genetic diagnosis, prenatal screening and carrier testing of familial and non-familial BBS patients.

## Background

Bardet Beidl syndrome (BBS) is a multi-symptomatic ciliopathy. The major clinical manifestations of this disorder are cone-rod dystrophy, truncal obesity, postaxial polydactyly, genital anomaly, learning disability and renal dysfunction [1]. Clinically BBS is a heterogeneous disorder with variable inter- and intra-familial features [2], therefore its diagnosis is established by the presence of four or three of the major features and two minor features like speech delay, brachydactyly/syndactyly, cardiovascular anomalies etc. [3, 4]. The incidence rate of BBS varies among the different ethnicities, and occur with the ratio of 1: 156,000 in Tunisia [5], 1: 140,000–160,000 in North America to Europe, 1: 17,000 in Kuwait, and 1: 18,000 in Newfoundland [6, 7].

Genetically BBS is segregated in an autosomal recessive fashion with high degree of locus and allele heterogeneity. The combinatorial approach of SNP microarray (for homozygosity mapping) with targeted exome sequencing (for mutation analysis) has speed-up the discovery of causative gene and its underlying pathogenic mutation [8, 9]. Genetic analysis of BBS patients have identified nineteen disease responsible genes (BBS1-19), most of which encode BBSome protein (*BBS1, BBS2, BBS4, BBS5, BBS7, BBS8/TTC8, BBS9/PTHB1,* BBS17/*LZTFL1* and *BBS18/BBIP1*) while some produce basal body interacting protein (BSS13/*MKS1, BBS14/EP290/NPHP6, BBS15/WDPCP* and *BBS16/SDCCAG8*), chaperonin complex protein (MKKS, BBS10 and BBS12), E3 ubiquitin ligase (BBS11/TRIM32), and a GTPase protein complex (*BBS3/ARL6* and *BBS19/IFT27*) [10, 11]. Physiologically BBSome is of prime importance in ciliogenesis due to its functional role in membrane trafficking in primary cilium [12]. The mutational spectrum of BBS genes is variable among different populations. A high proportion of European and Caucasian families are reported to have mutation in *BBS1* and *BBS10* gene with the ratio of 23.2 % and 20 % respectively [13, 14], while in the Arab population most of the mutations are found in *BBS1*, *BBS2, BBS3, BBS4* and *BBS8* [10, 15–17] with a total prevalence rate of 67 %. Although no prevalence data of the BBS genes have been estimated yet in the Pakistani population however most of the mutations occur in BBS1 [18], BBS3/ARL6 [19], BBS10 [19, 20], and BBS12 [21].

In the present linkage study, we have ascertained a Saraiki origin BBS family from the Kirikhaisor village of D.I.Khan in the KPK province of Pakistan. It was a consanguineous family with two affected siblings presenting with BBS features. Homozygosity mapping in combination with Sanger sequencing of a candidate gene identified a novel single base deletion mutation in *BBS9* gene [(c.299delC) & (p.Ser100Leufs*24)]. This frame shift mutation presumably leads to truncated nonfunctional protein. These data present the first report of the *BBS9* gene mutation in a Pakistani family and thus increase the mutational spectrum of Bardet Beidl syndrome in this ethnic region.

## Methods

### From sampling to DNA isolation

The consanguineous family for the present molecular investigation was ascertained from the rural area of D.I.Khan in the Khyber-Pakhtoonkhwa Province of Pakistan. The study was approved by the ethical review board of Gomal Centre of Biochemistry and Biotechnology, Gomal University D.I.Khan, Pakistan. Moreover, informed written consent was obtained from all individuals participating in the study. The family was visited at its residence and blood samples were collected. The patients were examined in detail for all apparent phenotypic features to document the major as well as the minor BBS symptoms. The parents were also counseled in detail on prenatal, perinatal, and neonatal medical problems. Subsiquently, the DNA was isolated from the peripheral blood sample by using standard Phenol-Chloroform method.

### From genome to gene analysis

To investigate the underlying disease gene mutation, genome-wide SNP genotyping was performed by using Affymetrix CytoScan 750 K SNP microarray. The SNP data were analyzed using the Chromosome Analysis Suite (ChAS) version 1.2 (Affymetrix) to identify the homozygous by descend region (HBD). The perfect segregation of identified disease associated HBD region in the complete family was determined by genotyping STR markers D7S493 (36.08 cM), D7S516 (42.93 cM), D7S484 (53.73 cM), and D7S510 (59.8 cM) {Rutgers map B37 [22]} within the homozygous region. For statistical significance of linkage, two point and multi point LOD scores were calculated by using Superlink and GeneHunter software packages plugged-in the EasyLinkage plus v5.02 software. The analysis was performed assuming a disease allele frequency of 0.001 with full penetrance at zero recombination fraction [23].

The primers for protein coding exons of *BBS9* gene were designed using the online version of Primer 3 plus software (version 0.4.0) [24]. The sequence reactions were performed with BigDye Terminator v3.1 cycle sequencing kit (Applied Biosystem, USA) and the sequencing products were analyzed by the Genetic analyzer 3130xl (Life Technologies, USA). The sequence data were then aligned with the *BBS9* reference sequence via blast like alignment tool (BLAT) tool package of UCSC Genome Browser [25].

## Results

**Fig. 1:**
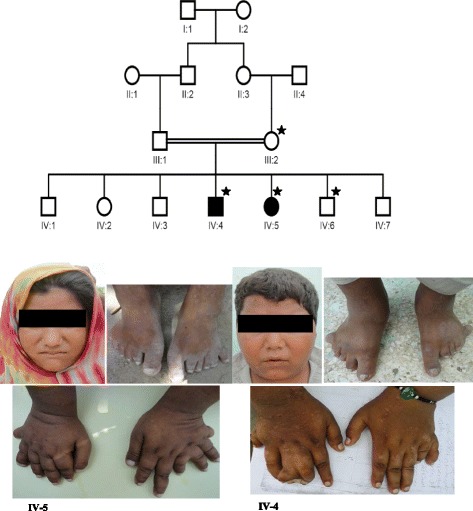
Family pedigree and facial photographs of patient IV-4 and IV-5. Symbols, tagged with asterisks, indicate individuals selected for genetic analysis in addition to both affected persons

In this study, four individuals from a multi-generation family were enrolled for molecular investigation of an underlying disease gene mutation. The recruited individuals consist of both affected siblings (IV-4 and IV-5), one unaffected brother (IV-6) and an asymptomatic carrier mother (III-2). Pedigree analysis revealed an autosomal recessive mode of disease segregation (see Fig. 1).

### Clinical findings

The clinical examination of both BBS patients demonstrated bilateral postaxial synpolydactyly of hands and feet, mild progressive retinal degeneration, intellectual disability, renal dysfunction, and obesity as a major BBS features, while mild hearing loss was also noticed as a minor feature in both affected individuals. The pattern of postaxial synpolydactyly in the female showed hepta-dactyly of left hand and foot but the right hand and foot had six digits, while the affected male had only hepta-dactyly of the right hand. The detailed clinical description of both affected individuals is shown in Table 1.

**Table 1:** Clinical description of BBS features presented by both patients

	Affected male (IV-4)	Affected female (IV-5)
Anthropometric data		
Age	20 Years	18 Years
Height	5 feet 2 inch	4 feet 10 inch
Weight	75 Kg	65 Kg
BMI^a^	30.2 (Obese)	30.9 (Obese)
Major BBS phenotypes		
Retinal degeneration	Yes	Yes
Polydactyly	Yes	Yes
Obesity	Yes	Yes
Developmental delay	Yes	Yes
Hypogonadism	No	No
Renal abnormality	Yes	Yes
Minor BBS phenotypes		
Speech disability	Yes	Yes
Strabismus, cataract, astigmatism	Yes	No
Brachydactyly, syndactyly	Yes	Yes
Diabetes mellitus	No	No
Ataxia, imbalance	No	No
Mild spasticity	No	No
Dental anomaly	No	No
Heart problems	No	No
Liver disorders	No	
Hearing Loss	No	Yes
Gastro-intestinal complications	Digestion problem	Digestion problem
Dermatologic issues	No	No
Menstruation in female	-	Irregular

^a^“BMI calculator” available on CDC (website www.cdc.gov) is used for BMI calculation

### Genetic findings

#### Whole genome SNP genotyping and linkage analysis

Genome-wide homozygosity mapping revealed a 18.45 Mb (HG19: chr7: 21,596,876-39,320,676) long HBD region between markers rs56356943 to rs12112084, on the short arm of chromosome 7. Segregation mapping by STR markers in the complete family, through STR markers, confirmed the inheritance of the homozygous region with disease etiology. Linkage analysis generated a suggestive two point and multipoint LOD scores of 0.92 and 1.26 respectively at same marker D7S484 (53.73 cM) with zero recombination fraction. The identified homozygous interval harbored the *BBS9* gene which is reported to be associated with BBS.

### Sequence analysis of the *BBS9* gene

**Fig. 2:**
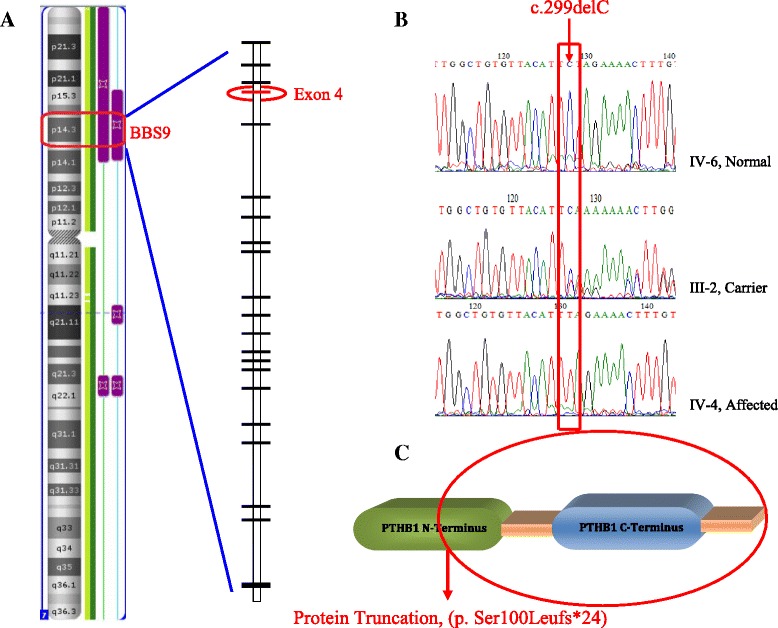
**a**- Autozygosity image, showing the common homozygous interval on 7p14.3 that harbors the known *BBS9* gene and the defect was detected in its exon 4. **b**- Sequence chromatogram of *BBS9* exon 4 is showing a homozygous deletion of cytosine in an affected individual (IV-4), heterozygous mutation in a carrier (III-2) and wild-type sequence in an normal individual (IV-6). **c**- The protein truncating mutation is depicting a loss of complete PTHB1 C-terminus domain along with some part of N-terminus, leaving behind a 122 amino acid truncated protein

Sanger DNA sequencing of the *BBS9* gene revealed a homozygous deletion of cytosine nucleotide in exon 4 (c.299delC). At protein level the reported deletion is predicted to result in a frame-shift mutation p.Ser100Leufs*24 (UniProtKB accession: Q3SYG4). The predicted premature stop codon presumably disrupts the open reading frame and results in a truncated polypeptide of 122 amino acids with complete loss of C-terminal PTHB1 domain (see Fig. 2). The identified sequence variant is not present in exome variant server (URL: http://exac.broadinstitute.org/), exome aggregation consortium (URL: http://evs.gs.washington.edu/EVS/), The Singapore Human Mutation and Polymorphism database (URL: http://shmpd.bii.a-star.edu.sg/) the Indian Genetic Disease database (URL: http://www.igdd.iicb.res.in/IGDD/home.aspx) and 100 normal controls from Pakistan (*n* = 50) and Arab population (*n* = 50).

## Discussion

Ciliopathies are a distinct group of genetic disorders which are caused by primary ciliary dysfunction with pleiotropic effect. Bardet Biedl syndrome is a rare autosomal recessive ciliopathic disorder characterized by retinal degeneration, intellectual disability, obesity, renal complications and polydactyly as predominant phenotypes. It is a heterogenetic disorder with 19 causative genes, whose protein products together constitute centrosome and/or basal body which is physiologically involved in ciliary function [26, 27]. Here in this report, we discovered a novel single base deletion in the 4th exon of *BBS9* gene (c.299delC), which presumably leads to truncated protein with complete loss of the C terminal PTHB1 domain (UniProtKB accession:Q3SYG4). The presence of the premature stop codon will likely lead to degradation of the resulting mRNA through Nonsense-mediated mRNA Decay.

BBS9 protein is the component of BBSome protein complex, a central entity of ciliogenesis, which is localized in the primary cilia and peri-centriolar region. Ten BBSome subunits (from BBS1 to 10) have been discovered, and BBS9 has a possible role in associating other subunits [12]. STRING protein interaction database (URL: www.string-db.org) predicted strong interaction of BBS9 with BBS5 protein, which suggests that BBS5 could be involved in the assembly of BBSome subunits through BBS9 interaction. Hence, loss of function mutation in BBS9 may affect the integrity of BBSome proteins complex due to a structural abnormality in cilia. This structural aberration could be the reason for disassembly of BBSome subunits due to defective BBS9-BBS5 protein interaction which might be required for proper organization. The knockdown studies in zebrafish and mouse have clearly demonstrated the significant role of BBS9 in cilia biogenesis [28]. Although extensive work has been performed for the genetic screening of BBS genes, but the actual function of most BBS proteins are yet to be discovered.

The mutational spectrum of *BBS9* gene has reported 12 missense/non-sense mutations, 2 splice site changes, 2 small deletions, 2 small insertions and 4 gross deletions in BBS phenotype (HGMD database; URL: http://www.hgmd.cf.ac.uk/ac/index.php), where as one complex rearrangement in *BBS9* is associated with Wilms tumor (HGMD database; [29]). The clinical spectrum of both patients reported in this study is consistent with previous reports, except for a few novel phenotypic variations. These clinical variations included digestion problems (constipation) in both affected (IV-4 & IV-5), irregular menstrual cycle (IV-5), strabismus (IV-4), and synpolydactyly of internal digits (IV-4 & IV-5).

The molecular genetics testing of any inherited disorder either involves multi-gene panel or the most implicated genes. In case of BBS, the high cost of multi-gene panels (BBS1- BBS19) and weakly established genotype-phenotype correlations favor the screening of highly prevalent BBS1 and BBS10 genes. Hence, keeping in view the genetic counseling of BBS patients, the large number of reported BBS genes and unclear phenotypic association data, genetic screening of the most implicated BBS1 and BBS10 is considered as cost effective. Nevertheless, prevalences of supposedly rare BBS genes such as BBS9 might vary in different ethicities and therefore prioritization of genes have to be adapted accordingly in a diagnostic setup.

In summary, our data represent the first *BBS9* gene mutation being reported in a Pakistani family associated with Bardet Biedl syndrome. In addition to cardinal features of BBS, slight clinical variations were also observed in Pakistani siblings. It is assumed that the current study will contribute to the elucidation of the genetic heterogeneity of BBS patients and that the screening of additional BBS cases in future might detect the mutational hotspot and thus would be helpful in designing a multi-gene panel for routine diagnosis.

## Conclusion

Here in this study we report the first Pakistani family segregating a novel deletion mutation in BBS9 gene (c.299delC). The mutation shifts the reading frame and presumably results in a truncated protein with loss of functional PTHB1 domains (p.Ser100Leufs*24). The current finding has increased the mutational spectrum of the Bardet Biedl Syndrome mutation in the Pakistani population and thereby will increase our knowledge to understand the potential role of the BBS9 protein in BBSome protein complex.

## Abbreviation

BBS: Bardet Biedl syndrome
D.I.Khan: Dera Ismail Khan
KPK: Khyber-Pakhtoonkhwa
DNA: Deoxy ribonucleic acid
SNP: Single nucleotide polymorphism
ChAS: Chromosome analysis suite
HBD: Homozygous by Descend
cM: Centi Morgan
LOD: Logarithm of odd
BLAT: Blast like alignment tool
Mb: Mega base
STR: Short tandem repeat
Ser: Serine
Leu: Leucine
fs: Frame shift
HGMD: Human gene mutation database
